# 38例肺肉瘤样癌临床特点及预后的回溯性分析

**DOI:** 10.3779/j.issn.1009-3419.2015.09.02

**Published:** 2015-09-20

**Authors:** 园园 李, 丽丽 张, 娟 蒋, 华平 杨, 立明 曹, 其华 顾, 敏 李, 成平 胡

**Affiliations:** 410008 长沙，中南大学湘雅医院呼吸科 Department of Respiratory Medicine, Xiangya Hospital of Central South University, Changsha 410008, China

**Keywords:** 肺肉瘤样癌, 临床特点, 生物靶向治疗, 手术, 远处转移, 预后, Pulmonary sarcomatoid carcinoma, Clinical characteristics, Biological targeted therapy, Surgery, Distant metastasis, Prognosis

## Abstract

**背景与目的:**

肺肉瘤样癌（pulmonary sarcomatoid carcinoma, PSC）是一种罕见的肺部恶性肿瘤，属于非小细胞癌。但因其发病率低，目前治疗方法尚无定论。本研究旨在探讨PSC的临床特点、诊断标准、治疗方法及预后，为临床诊治提供一定依据。

**方法:**

回顾性分析2000年1月-2013年12月在我院治疗的38例肺肉瘤样癌患者的一般临床资料、吸烟史、临床表现、肿瘤大小、TNM分期、病理与免疫组化结果、诊断方法、治疗方法及预后。使用SPSS 19.0统计软件对结果进行分析，生存期使用*Kaplan-Meier*法进行分析。

**结果:**

本组患者年龄26岁-76岁（中位年龄57.5岁），男女比例约为4:1，81.6%的患者有长期大量吸烟史。咳嗽和咯血是最常见的首发症状。全部患者的中位生存期为21个月。1年、3年、5年累计生存率分别为68.4%、31.6%和18.4%。肿瘤大小、TNM分期、肿瘤是否发生远处转移及是否进行手术与患者的预后相关。

**结论:**

肺肉瘤样癌临床表现无特殊，影响肺肉瘤样癌预后的主要因素有：肿瘤大小、TNM分期、肿瘤是否发生远处转移及是否进行手术。目前，手术根治切除是治疗PSC的关键，其放化疗的综合治疗方式与配伍还需开展更多循证医学的探索。生物靶向药物可为PSC患者的治疗提供新的方向。

## Abstract (en)

**Background and objective:**

Pulmonary sarcomatoid carcinoma is a rare histologic subtype of non-small cell lung cancer. The effective treatment for this disease has not well defined due to its extremely low morbidity. This study explores the clinicopathological characteristics and prognosis of 38 patients with PSC, so as to provide some clues for its diagnosis and treatment.

**Methods:**

The study enrolled 38 patients with PSC that were diagnosed with histology or cytology in our hospital between January 2000 and December 2013. We retrospectively analyzed general clinical characteristics, smoking history, tumor size, TNM staging, pathology, immunohistochemistry, diagnostic method, treatment and prognosis. We used SPSS 19.0 statistical software and *Kaplan-Meier* method to analyze our data.

**Results:**

Patients in this study were aged from 26 to 76 years old (the median age was 57.5 years old). Among all of them, the male to female ratio was 4:1, and 81.6% of patients had smoking history. Cough and hemoptysis were the most common primary symptoms. The median survival was 21 months, while one-year survival rate, three-year survival rate and five-year survival rate were 68.4%, 31.6% and 18.4% respectively. Tumor size, TNM staging, distant metastasis and surgery therapy were associated with the prognosis of patients.

**Conclusion:**

Patients with PSC present with no special symptoms generally. According to our study, factors that affect patients' prognosis include tumor size, TNM staging, distant metastasis and surgery. Complete resection is the key treatment for PSC patients, but comprehensive chemoradiotherapy needs further exploration in evidence-based medicine. Biological target therapy may give new insight into treatment for PSC.

肺肉瘤样癌（pulmonary sarcomatoid carcinoma, PSC）是一种很罕见的肺癌，约占所有肺癌类型的4.7%^[1]^。2004年世界卫生组织（World Health Organization, WHO）将PSC定义为含有肉瘤及肉瘤样成分的预后较差的非小细胞肺癌（non-small cell lung cancer, NSCLC）^[2]^。由于肺肉瘤样癌较为罕见，故与其他常见的NSCLC相比，其临床特点、诊断方法及治疗手段还存在诸多争议。本研究将我院38例PSC患者的临床特点及预后资料进行分析，以期为临床诊治提供依据。

## 1 资料与方法

### 1.1 患者与资料

收集湘雅医院2000年1月-2013年12月诊断的38例PSC患者，所有患者均经病理学与免疫组化检测确诊，且临床资料完整。

收集所有患者的以下数据：一般临床资料、吸烟史、临床症状、肿瘤大小、临床分期、病理及免疫组化结果、诊断方法、治疗方法及预后。其中吸烟史统计有无吸烟史以及吸烟指数（定义为每天吸烟包数乘以吸烟年数）。肿瘤分期标准采用国际抗癌联盟（International Union Against Cancer, UICC）的TNM分期（第7版）。

### 1.2 统计学方法

使用SPSS 19.0统计软件进行分析。生存期使用*Kaplan-Meier*法进行分析。*P* < 0.05为差异有统计学意义。

## 2 结果

### 2.1 临床特点（表 1）

**1:** 38例PSC患者的临床特点 Clinical characteristics of the 38 patients with PSC

Characteristics	*n*	Proportion (%)
Gender		
Male	31	81.6
Female	7	18.4
Age(year)		
≤60	24	63.6
> 60	14	36.8
Smoking history		
Yes	31	81.6
No	7	18.4
Clinical symptoms		
Cough	24	63.2
Hemoptysis	17	44.7
Chest pain	7	18.4
Fever	6	15.8
Fatigue	4	10.5
Emaciation	3	7.9
TNM staging		
Ⅰ	6	15.8
Ⅱ	19	50.0
Ⅲ	2	5.3
Ⅳ	11	28.9
Metastasis		
Lymphnode metastasis	11	28.9
Distant metastasis	12	31.6
Immunohistochemistry		
Cytokeratin	31	81.6
Vimentin	36	94.7
EMA	18	47.4
TTF-1	8	21.1
CEA	6	15.8
PSC: pulmonary sarcomatoid carcinoma; EMA: epithelial membrane antigen; TTF-1: thyroid transcription factor-1; CEA: carcinoembryonic antigen.		

#### 2.1.1 一般情况

本组38例患者中，男性31例，女性7例。患者年龄范围26岁-76岁，中位年龄为57.5岁。

#### 2.1.2 吸烟史

31例患者（81.6%）有长期大量吸烟史（吸烟指数≥400年支）。

#### 2.1.3 临床症状

所有患者的临床表现大多无特异性，最常见的临床症状包括：咳嗽（24例，63.2%）、咯血（17例，44.7%）、胸痛（7例，18.4%）；呼吸系统以外的症状包括：发热（6例，15.8%）、乏力（4例，10.5%）、消瘦（3例，7.9%）；另外有2例无任何症状，体检发现肺部肿块。

#### 2.1.4 肿瘤大小

肿瘤最大直径3.0 cm-14.0 cm，中位值为6.0 cm。肿瘤均经我院病理科病理组织学检查确诊为肺肉瘤样癌。

#### 2.1.5 临床分期和转移部位

诊断时，11例患者（28.9%）出现了肺内、胸膜、纵隔、肝或骨的远处转移；12例患者（31.6%）出现淋巴结转移；诊断时各患者的肺癌分期为Ⅰ期患者6例，Ⅱ期19例，Ⅲ期2例，Ⅳ期11例。

### 2.2 诊断方法

经手术标本活检确诊25例（65.8%）、经计算机断层扫描（computed tomography, CT）/B超引导下肺穿刺活检10例（26.3%）、经电子支气管镜下活检确诊3例（7.9%）。

### 2.3 病理与免疫组化结果

本研究中38例PSC患者均行病理与免疫组化检查（图 1）。其中，上皮样标志物Cytokeratin阳性率为81.6%（31/38），间叶细胞标志物Vimentin阳性率为94.7%（36/38），EMA阳性率为47.4%（18/38），甲状腺转录因子1（thyroid transcription factor, TTF-1）阳性率为21.1%（8/38），癌胚抗原（carcino-embryonic antigen, CEA）阳性率为15.8%（6/38）。此外，本研究中共有5例患者进行了表皮生长因子受体（epidermal growth factor receptor, *EGFR*）基因检测，其中有1例为EGFR基因突变阳性，阳性率为20.0%（1/5）。

**1:**
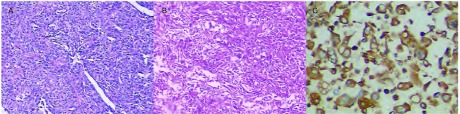
病理与免疫组化检查。A：肿瘤组织内可见巨大肿瘤细胞（HE染色，×100）；B：肿瘤细胞呈梭形细胞肉瘤样改变（HE染色，×100）；C：免疫组化标记Vimentin呈阳性表达（×400） Pathology and immunohistochemistry. A: Giant tumor cells could be seen in tumor tissue (H & E staining, ×100); B: Tumor cells showing sarcomatoid changes with atypical pindle cells (H & E staining, ×100); C: Immunohistochemical staining with Viemntin (×400)

### 2.4 治疗方法

根据患者PSC的临床分期、美国东部肿瘤协作组（Eastern Cooperative Oncology Group, ECOG）评分及主治医师的临床经验制定综合治疗方案。本组患者中，32例患者行手术治疗。其中，28例为根治性手术，3例为姑息性手术，还有1例患者开胸后发现右上肺肿瘤侵犯胸壁，且肺门纵隔淋巴结肿大并包绕肺门，固定成“冰冻”状，无法解剖肺门结构，故无法切除。根治性手术中单纯肺叶切除18例（64.3%），全肺切除6例（21.4%），肺叶+肺段切除2例（7.1%），肺叶+肺动脉干侧壁切除2例（7.1%）。28例患者均行纵隔淋巴结清扫。在3例行姑息性手术的患者中，2例行楔形切除+部分胸壁切除，另1例行肺叶切除+部分胸壁切除。9例患者在手术治疗后3周内进行辅助化疗，其中，5例采用TP方案（紫杉醇+顺铂），2例采用IAP方案（异环磷酰胺+表阿霉素+顺铂），2例采用DP方案（多西他赛+萘达铂）。9例患者行单纯化疗，其中，5例采用TP方案，3例采用IAP方案，1例采用DP方案。值得注意的是，本研究中有1例患者EGFR基因突变阳性，口服吉非替尼靶向药物治疗后症状消失，病灶稳定4个月。

### 2.5 随访

在随访终止时，30例患者死亡，5例存活，3例失访。38例患者的生存期为3个月-118个月，中位生存期为21个月。其1年、3年、5年累计生存率分别为68.4%、31.6%、18.4%（图 2）。肿瘤直径为 < 3 cm、3 cm-7 cm、 > 7 cm组患者中位生存期分别为49个月、25个月、8个月（图 3A）；Ⅰ期、Ⅱ期、Ⅲ期、Ⅳ期患者中位生存期分别为48个月、32个月、13个月及7个月（图 3B）；存在转移及未出现转移患者的中位生存期分别7个月、38个月（图 3C）；进行根治性手术及未进行手术治疗患者的中位生存期分别为38个月、8个月（图 3D）。以上各组内差异均有统计学意义（*P* < 0.05）。

**2:**
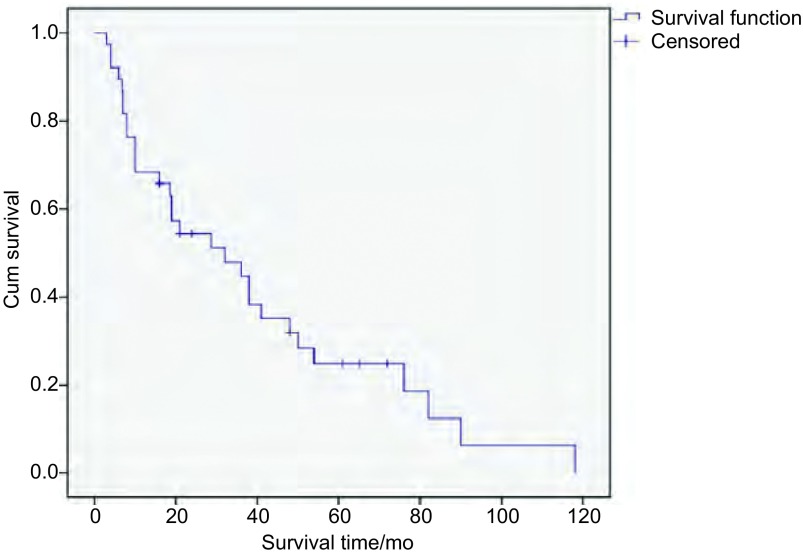
所有肺肉瘤样癌患者的总生存分析 Overall survival of all PSC patients

**3:**
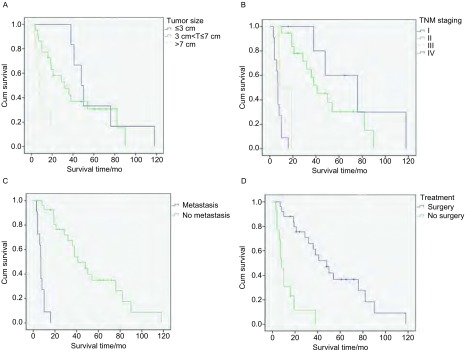
不同临床因素对肺肉瘤样癌患者生存曲线的影响。A：肿瘤大小；B：TNM分期；C：远处转移；D：手术治疗 Effects of different factors on PSC patients'survival curve. A: Tumor size; B: TNM staging; C: Distant metastasis; D: Surgery

## 3 讨论

PSC系一种发生率较低的NSCLC，多见于吸烟的老年男性患者^[3]^。本研究中患者的年龄在26岁-76岁之间，中位年龄为57.5岁，其中大部分患者有长期大量吸烟史（81.6%），表明吸烟可能与肺肉瘤样癌的肿瘤形成相关，但有无吸烟史对患者的预后并无统计学意义。类似地，其他研究^[4, 5]^也表明吸烟史对PSC患者的总体生存期并无明显影响。

PSC患者无特异性临床表现，其症状与肿瘤部位有一定关系，以咳嗽与咯血多见。其诊断依赖于镜下癌细胞病理形态及免疫组织化学染色。电镜下观察肉瘤样细胞具有上皮细胞特征，如果仅含有肉瘤样成分则需要进行免疫组化标记以证实其上皮样分化方可诊断^[6, 7]^。PSC同时含有上皮与间质成分，不同的组织成分常掺杂在一起，并有移行过渡区，PSC是由上皮癌组织通过上皮间质转化（epithelial-mesenchymal transition, EMT）而逐渐形成。这种表型转换使肿瘤细胞摆脱细胞间连接的束缚，而表现出更强的侵袭性，对PSC的浸润和转移起着至关重要的作用。所以上皮标记的减少和间叶标记的增多往往提示着患者预后较差及对EGFR-酪氨酸激酶抑制剂（tyrosine kinase inhibitor, TKI）靶向治疗耐药性的形成。近年来，研究^[3]^已证实对EMT的负性调控是肉瘤样癌的治疗靶点之一，可能对其发展、转移产生一定程度的抑制。在本研究中，大部分患者肉瘤样组织都同时表达了上皮样标志物（如31例/38例患者细胞角蛋白Cytokeratin阳性表达）及间叶细胞标志物（如36例/38例患者波形蛋白Vimentin阳性表达）。由于PSC组织成分的多样性，部分PSC患者会出现*EGFR*基因突变、*K-RAS*基因突变或者*EML4-ALK*基因融合。有学者^[8]^报道，约有28.1%的PSC患者*EGFR*基因突变阳性，3%的患者*K-RAS*基因突变阳性。在本组患者中，共有5例患者进行了*EGFR*基因检测，其中有1例患者为*EGFR*基因突变阳性，并服用吉非替尼靶向药物进行治疗。治疗后该患者的临床症状消失，肿瘤稳定4个月。此外，也有研究^[4]^表明，*K-RAS*基因突变比*EGFR*基因突变更常见（约为38%），但我们并未对患者进行*K-RAS*基因的检测。我们分析，PSC患者出现*EGFR*基因或*K-RAS*基因突变的主要原因是其表皮样组织的基因突变，但还需要进一步的基础及临床研究予以证实。突变型PSC患者的生物靶向治疗也需要更多的临床研究进行探索。

PSC的治疗方式目前是以手术为主、放化疗为辅的综合治疗。由于PSC较为罕见，目前其化疗方案是参照NSCLC的化疗方案进行，但治疗效果不佳^[9]^。部分研究^[5, 10]^表明，包含术后辅助化疗的综合治疗方案可提高患者生存率，然而，PSC患者的辅助化疗有效性还存在争议。本研究中，9例患者在手术治疗后3周内进行辅助化疗，9例患者行单纯化疗治疗，但这些患者的生存期相较未行辅助化疗的手术患者的生存期并无明显统计学差异。这与某些国外的报道^[11, 12]^结果相似，可能是由肿瘤大小差异、样本量较小或者其他因素造成。到目前为止，关于放化疗是否可以预防PSC转移和复发的研究很少。还有研究^[13]^表明，晚期PSC患者对化疗的反应率很低。因此，PSC患者的有效治疗方案仍需要进行更多大样本的临床前瞻性对照研究，以EMT为靶点的生物靶向治疗可能在未来取得突破。

本研究中，肿瘤的大小明显影响生存率，而淋巴结转移情况对患者预后无明显影响。PSC患者出现远处转移提示其预后不良，这与已有报道结果^[14]^相一致。肉瘤样癌的成分与肿瘤大小相关，当肿瘤直径较小时，其肉瘤样成分也较少，这可能是导致其预后明显好于直径较大的肉瘤样癌患者的重要原因之一。Yuki等^[15]^研究发现，早期PSC患者即可有大部分（57.1%）出现血管的侵犯，这解释了为何有患者在患病早期就出现了远处转移，也说明了淋巴结转移状态不影响预后的原因。本组患者的5年生存率为18.4%，与已有报道结果相近；对于早期PSC患者来说相对较低；中位生存期21个月，相对于其他研究^[4, 11]^的数据稍高。这可能与本研究中Ⅰ期与Ⅱ期患者较多，且绝大多数患者都进行了根治性手术治疗有关。

肺肉瘤样癌的临床表现无特殊，影响其预后的主要因素有：肿瘤大小、TNM分期、肿瘤是否发生远处转移及是否进行手术。手术根治切除是治疗局部PSC的关键措施，其放化疗的综合治疗方式还需开展更多的循证医学的探索，生物靶向药物可为PSC患者的治疗提供新的方向。
